# Ethyl 2,7,7-trimethyl-4-(1-methyl-1*H*-indol-3-yl)-5-oxo-1,4,5,6,7,8-hexa­hydro­quinoline-3-carboxyl­ate

**DOI:** 10.1107/S1600536812047976

**Published:** 2012-12-08

**Authors:** Sema Öztürk Yildirim, Ray J. Butcher, Miyase Gözde Gündüz, Ahmed El-Khouly, Rahime Şimşek, Cihat Şafak

**Affiliations:** a Department of Chemistry, Howard University, 525 College Street NW, Washington, DC 20059, USA; b Department of Physics, Faculty of Sciences, Erciyes University, 38039 Kayseri, Turkey; c Hacettepe University, Faculty of Pharmacy, Dept. of Pharmaceutical Chemistry, 06100 Sihhiye-Ankara, Turkey

## Abstract

In the title mol­ecule, C_24_H_28_N_2_O_3_, the cyclo­hexene ring is in a sofa conformation and the 1,4-dihydro­pyridine ring is in a slight boat conformation. In the indole ring system, the pyrrole and benzene rings form a dihedral angle of 2.63 (7)°. In the crystal, N—H⋯O hydrogen bonds connect the mol­ecules into *C*(6) chains parallel to the *b* axis and pairs of weak C—H⋯O hydrogen bonds link inversion-related chains into a ladder motif through *R*_2_^2^(18) rings. A weak intra­molecular C—H⋯O hydrogen bond is also observed.

## Related literature

For the biological functions of calcium ions, see: Triggle & Swamy (1980 ▶) and for the biological functions and physiological roles of calcium channels, see: Zamponi (1997 ▶); Dolphin (2006 ▶). For the biological properties of 1,4-dihydro pyridines (DHP), see: Vaghy *et al.* (1987 ▶); Triggle (2003 ▶); Şafak & Şimşek (2006 ▶); Zhou *et al.*, (2011 ▶). For nifedipine (the prototypical DHP) in clinical use, see: Gordeev *et al.* (1998 ▶). For geometric analysis, see: Cremer & Pople (1975 ▶). For hydrogen-bond motifs, see: Bernstein *et al.* (1995 ▶). For similar structures, see: El-Khouly *et al.* (2012 ▶); Öztürk Yildirim *et al.* (2012 ▶); Gündüz, *et al.* (2012 ▶).
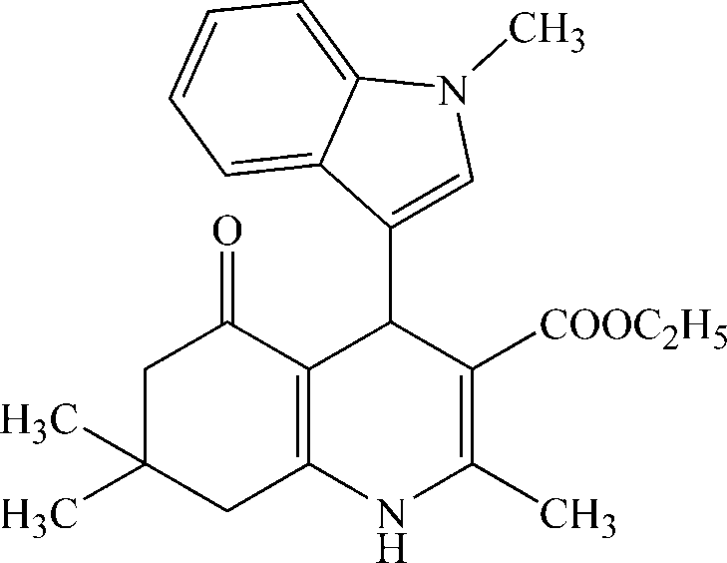


## Experimental

### 

#### Crystal data

C_24_H_28_N_2_O_3_
*M* *_r_* = 392.48
Monoclinic, 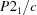

*a* = 17.4656 (4) Å
*b* = 10.1883 (2) Å
*c* = 12.3465 (3) Å
β = 106.806 (2)°
*V* = 2103.16 (8) Å^3^
*Z* = 4
Cu *K*α radiation
μ = 0.65 mm^−1^
*T* = 123 K
0.55 × 0.40 × 0.35 mm

#### Data collection

Agilent Xcalibur (Ruby, Gemini) diffractometer
Absorption correction: multi-scan [*CrysAlis RED* (Agilent, 2011 ▶), based on expressions derived from Clark & Reid (1995 ▶)] *T*_min_ = 0.715, *T*_max_ = 0.804
8035 measured reflections
4246 independent reflections
3533 reflections with *I* > 2σ(*I*)
*R*_int_ = 0.025

#### Refinement

*R*[*F* ^2^ > 2σ(*F* ^2^)] = 0.044
*wR*(*F* ^2^) = 0.125
*S* = 1.03
4246 reflections
271 parameters
H atoms treated by a mixture of independent and constrained refinement
Δρ_max_ = 0.27 e Å^−3^
Δρ_min_ = −0.23 e Å^−3^

### 

Data collection: *CrysAlis PRO* (Agilent, 2011 ▶); cell refinement: *CrysAlis PRO*; data reduction: *CrysAlis PRO*; program(s) used to solve structure: *SHELXS97* (Sheldrick, 2008 ▶); program(s) used to refine structure: *SHELXL97* (Sheldrick, 2008 ▶); molecular graphics: *SHELXTL*; software used to prepare material for publication: *SHELXTL*.

## Supplementary Material

Crystal structure: contains datablock(s) I, global. DOI: 10.1107/S1600536812047976/lh5560sup1.cif

Structure factors: contains datablock(s) I. DOI: 10.1107/S1600536812047976/lh5560Isup2.hkl

Additional supplementary materials:  crystallographic information; 3D view; checkCIF report

## Figures and Tables

**Table 1:** Hydrogen-bond geometry (Å, °)

*D*—H⋯*A*	*D*—H	H⋯*A*	*D*⋯*A*	*D*—H⋯*A*
C21—H21*A*⋯O2	0.98	2.28	2.8073 (19)	113
N1—H1*N*⋯O1^i^	0.86 (2)	1.98 (2)	2.8161 (15)	163.9 (19)
C24—H24*C*⋯O2^ii^	0.98	2.60	3.1693 (19)	118

Symmetry codes: (i) 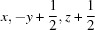; (ii) 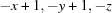.

**Fig. 1.**
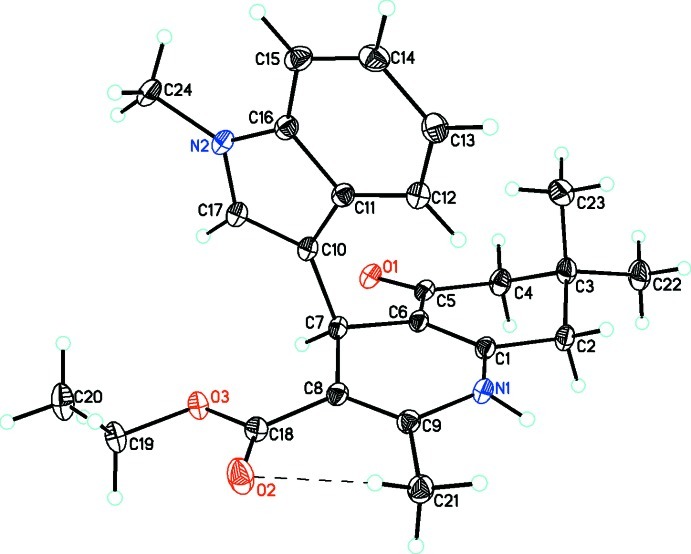
The molecular structure of (I), showing 30% probability displacement ellipsoids. The dashed line indicates the intramolecular C—H···O interaction.

**Fig. 2.**
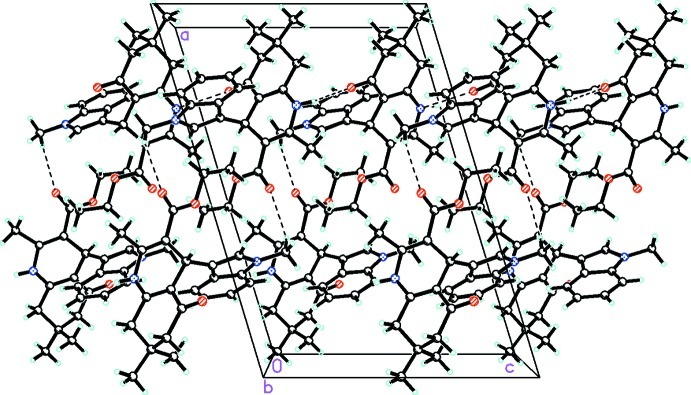
The packing and hydrogen bonding of the title molecule in the unit cell, viewing down b axis. Hydrogen bonds and C—H···O interactions are shown as dashed lines.

## supplementary crystallographic information

### Comment

Calcium ions play a critical role in various biological functions such as muscle contraction, release of neurotransmitters and regulation of neuronal excitability (Triggle & Swamy, 1980). Calcium entry into the cytosol is mediated by different types of calcium channels with distinct physiological roles (Zamponi, 1997). *L*-type channels are confined to cell bodies and regulate contractions in muscle cells. Calcium channel antagonists reversibly block Ca^2+^ influx through *L*-type calcium channels (Dolphin, 2006). 1,4-Dihydropyridines (DHP), of which nifedipine is the prototype, are one of the known classes of calcium antagonists which are frequently used for the treatment of cardiovascular diseases like angina, hypertension and supraventricular tachycardia (Vaghy *et al.*, 1987; Triggle, 2003; Şafak & Şimşek, 2006). DHPs have attracted interest since their introduction into clinical medicine, because of their high potency and selectivity of action (Zhou *et al.*, 2011). Many modifications have been carried out on the structure of nifedipine in order to enhance calcium modulating effects and lead to new active compounds (Gordeev *et al.*, 1998). According information obtained from structure-activity relationships and our experience in this field, we synthesized ethyl 2,7,7-trimethyl-4-(1-methyl-1*H*-indol-3-yl)-5-oxo-1,4,5,6,7,8-hexahydroquinoline-3-carboxylate and determined its crystal structure.

The molecular structure of the title compound is shown in Fig. 1. The (C1—C6) cyclohexene ring is in a sofa conformation with puckering parameters (Cremer & Pople, 1975) of Q_T_ = 0.492 (1) Å, θ = 120.6 (1) ° and φ = 302.2 (1)°. The 1,4-dihydropyridine ring (N1/C1/C6—C9) is in a slight boat conformation. In the 1*H*-indole ring system, the 2,3-dihydro-1*H*-pyrrole and benzene rings form a dihedral angle of 2.63 (7)°. The values of the bond lengths and bond angles are comparable with those of the related structures previously reported (El-Khouly *et al.*, 2012; Öztürk Yildirim *et al.*, 2012; Gündüz, *et al.*, 2012).

In the crystal, N—H···O hydrogen bonds connect molecules *via* C(6) motifs (Bernstein *et al.* 1995) into chains parallel to the *b* axis and pairs weak C—H···O hydrogen bonds link inversion related chains into a ladder motif through *R*_2_^2^(18) rings (Fig. 2). A weak intramolecular C—H···O hydrogen bond is also observed.

### Experimental

The compound was prepared by refluxing 5,5-dimethyl-cyclohexane-1,3-dione (0.001 mol), ethyl acetoacetate (0.001 mol), 1-methyl-3-indolecarbaldehyde (0.001 mol) and ammonium acetate (0.005 mol) in methanol for 8 h. After cooling, the mixture was poured into ice-water. The obtained precipitate was crystallized from ethanol (m.p. 507 K). Pure crystals suitable for X-ray structure analysis were obtained by slow evaporation method using methanol as a solvent. Its structure was elucidated by IR, ^1^H-NMR and elemental analysis. IR (cm^-1^): 3288, 3072, 2970, 1685; ^1^H-NMR δ (p.p.m.) 0.9–1.0 (6H; s; 2xCH_3_), 1.1 (3H; t; CH_2_CH_3_), 1.9–2.2 (4H; m; quinoline H^6,8^), 2.3 (3H; s; CH_3_), 3.6 (3H; s; N—CH_3_), 3.9 (2H; m; CH_2_CH_3_), 5.0 (1H; s; quinoline H_4_), 6.8 (1H; s; indole H^2^), 6.9–7.6 (4H; m; aromatic), 9.2 (1H; s; NH). Anal. for C_24_H_28_N_2_O_3_ calculated: C, 73.44; H, 7.19; N, 7.14; found: C, 74.05; H, 6.82; N, 7.41. The title compound demonstrated calcium channel blocker activity in isolated rat ileum and lamb carotid artery.

### Refinement

The N-bound H1N atom was located in a difference map and refined freely [N—H = 0.86 (2) Å]. The remaining H atoms were positioned geometrically (C—H = 0.95–1.00 Å) and allowed to ride on their parent atoms, with *U*_iso_(H) = 1.2*U*_eq_(C) or 1.5*U*_eq_(C_methyl_). A rotating group model was used for methyl groups.

### Crystal data

**Table:**

C_24_H_28_N_2_O_3_	*F*(000) = 840
*M**_r_* = 392.48	*D*_x_ = 1.240 Mg m^−^^3^
Monoclinic, *P*2_1_/*c*	Cu *K*α radiation, λ = 1.54184 Å
Hall symbol: -P 2ybc	Cell parameters from 3252 reflections
*a* = 17.4656 (4) Å	θ = 3.7–75.7°
*b* = 10.1883 (2) Å	µ = 0.65 mm^−^^1^
*c* = 12.3465 (3) Å	*T* = 123 K
β = 106.806 (2)°	Block, colorless
*V* = 2103.16 (8) Å^3^	0.55 × 0.40 × 0.35 mm
*Z* = 4	

### Data collection

**Table:**

Agilent Xcalibur (Ruby, Gemini) diffractometer	4246 independent reflections
Radiation source: Enhance (Cu) X-ray Source	3533 reflections with *I* > 2σ(*I*)
Graphite monochromator	*R*_int_ = 0.025
Detector resolution: 10.5081 pixels mm^-1^	θ_max_ = 75.9°, θ_min_ = 5.1°
ω scans	*h* = −20→21
Absorption correction: multi-scan [*CrysAlis RED* (Agilent, 2011), based on expressions derived from Clark & Reid (1995)]	*k* = −12→10
*T*_min_ = 0.715, *T*_max_ = 0.804	*l* = −14→15
8035 measured reflections	

### Refinement

**Table:**

Refinement on *F*^2^	Primary atom site location: structure-invariant direct methods
Least-squares matrix: full	Secondary atom site location: difference Fourier map
*R*[*F*^2^ > 2σ(*F*^2^)] = 0.044	Hydrogen site location: inferred from neighbouring sites
*wR*(*F*^2^) = 0.125	H atoms treated by a mixture of independent and constrained refinement
*S* = 1.03	*w* = 1/[σ^2^(*F*_o_^2^) + (0.0707*P*)^2^ + 0.4192*P*] where *P* = (*F*_o_^2^ + 2*F*_c_^2^)/3
4246 reflections	(Δ/σ)_max_ = 0.001
271 parameters	Δρ_max_ = 0.27 e Å^−^^3^
0 restraints	Δρ_min_ = −0.23 e Å^−^^3^

### Special details

**Table:**

Experimental. Absorption correction: CrysAlis RED, (Agilent, 2011) Empirical absorption correction using spherical harmonics, implemented in SCALE3 ABSPACK scaling algorithm. (Clark & Reid, 1995).
Geometry. All e.s.d.'s (except the e.s.d. in the dihedral angle between two l.s. planes) are estimated using the full covariance matrix. The cell e.s.d.'s are taken into account individually in the estimation of e.s.d.'s in distances, angles and torsion angles; correlations between e.s.d.'s in cell parameters are only used when they are defined by crystal symmetry. An approximate (isotropic) treatment of cell e.s.d.'s is used for estimating e.s.d.'s involving l.s. planes.
Refinement. Refinement of *F*^2^ against ALL reflections. The weighted *R*-factor *wR* and goodness of fit *S* are based on *F*^2^, conventional *R*-factors *R* are based on *F*, with *F* set to zero for negative *F*^2^. The threshold expression of *F*^2^ > σ(*F*^2^) is used only for calculating *R*-factors(gt) *etc*. and is not relevant to the choice of reflections for refinement. *R*-factors based on *F*^2^ are statistically about twice as large as those based on *F*, and *R*- factors based on ALL data will be even larger.

### Fractional atomic coordinates and isotropic or equivalent isotropic displacement parameters (Å^2^)

**Table:**

	*x*	*y*	*z*	*U*_iso_*/*U*_eq_	
O1	0.80036 (6)	0.10674 (11)	0.14843 (8)	0.0291 (2)	
O2	0.50682 (7)	0.45404 (15)	0.20829 (11)	0.0444 (3)	
O3	0.53584 (6)	0.31811 (11)	0.08329 (9)	0.0281 (2)	
N1	0.74838 (7)	0.40267 (13)	0.41035 (10)	0.0250 (3)	
N2	0.70297 (7)	0.49919 (13)	−0.07455 (10)	0.0254 (3)	
C1	0.79738 (8)	0.31973 (14)	0.37441 (11)	0.0235 (3)	
C2	0.87530 (9)	0.28770 (16)	0.46042 (12)	0.0289 (3)	
H2A	0.8662	0.2185	0.5116	0.035*	
H2B	0.8952	0.3668	0.5066	0.035*	
C3	0.93922 (9)	0.24060 (16)	0.40645 (12)	0.0301 (3)	
C4	0.90135 (9)	0.13072 (16)	0.32375 (13)	0.0312 (3)	
H4A	0.9397	0.1037	0.2825	0.037*	
H4B	0.8921	0.0540	0.3675	0.037*	
C5	0.82268 (8)	0.16811 (14)	0.23802 (11)	0.0240 (3)	
C6	0.77439 (8)	0.26933 (14)	0.26782 (11)	0.0221 (3)	
C7	0.69728 (8)	0.31300 (14)	0.18198 (11)	0.0218 (3)	
H7A	0.6704	0.2333	0.1409	0.026*	
C8	0.64109 (8)	0.37442 (14)	0.24284 (11)	0.0232 (3)	
C9	0.66851 (8)	0.42081 (14)	0.34996 (12)	0.0242 (3)	
C10	0.71174 (8)	0.40744 (14)	0.09521 (11)	0.0223 (3)	
C11	0.75097 (8)	0.53337 (14)	0.11377 (12)	0.0233 (3)	
C12	0.78991 (8)	0.60792 (15)	0.20974 (12)	0.0269 (3)	
H12A	0.7970	0.5736	0.2835	0.032*	
C13	0.81773 (9)	0.73194 (16)	0.19525 (13)	0.0308 (3)	
H13A	0.8438	0.7827	0.2599	0.037*	
C14	0.80824 (9)	0.78432 (16)	0.08691 (14)	0.0317 (3)	
H14A	0.8277	0.8700	0.0796	0.038*	
C15	0.77111 (9)	0.71327 (16)	−0.00918 (13)	0.0297 (3)	
H15A	0.7647	0.7485	−0.0825	0.036*	
C16	0.74337 (8)	0.58779 (15)	0.00536 (12)	0.0249 (3)	
C17	0.68333 (8)	0.39281 (14)	−0.01990 (11)	0.0246 (3)	
H17A	0.6540	0.3189	−0.0568	0.029*	
C18	0.55550 (8)	0.38803 (15)	0.18007 (12)	0.0266 (3)	
C19	0.45226 (9)	0.32978 (17)	0.01690 (13)	0.0317 (3)	
H19A	0.4382	0.4232	0.0000	0.038*	
H19B	0.4171	0.2937	0.0598	0.038*	
C20	0.44107 (10)	0.2548 (2)	−0.09068 (15)	0.0436 (4)	
H20A	0.3850	0.2606	−0.1364	0.065*	
H20B	0.4553	0.1626	−0.0731	0.065*	
H20C	0.4755	0.2920	−0.1330	0.065*	
C21	0.62218 (9)	0.49093 (17)	0.41785 (13)	0.0313 (3)	
H21A	0.5844	0.5522	0.3688	0.047*	
H21B	0.6592	0.5396	0.4796	0.047*	
H21C	0.5927	0.4267	0.4494	0.047*	
C22	1.01088 (10)	0.1881 (2)	0.49980 (15)	0.0404 (4)	
H22A	1.0511	0.1540	0.4659	0.061*	
H22B	0.9931	0.1174	0.5408	0.061*	
H22C	1.0342	0.2592	0.5525	0.061*	
C23	0.96694 (10)	0.35270 (19)	0.34418 (15)	0.0390 (4)	
H23A	1.0071	0.3197	0.3095	0.058*	
H23B	0.9904	0.4227	0.3980	0.058*	
H23C	0.9211	0.3874	0.2852	0.058*	
C24	0.67642 (9)	0.52313 (17)	−0.19626 (12)	0.0307 (3)	
H24A	0.6508	0.4438	−0.2354	0.046*	
H24B	0.7226	0.5459	−0.2225	0.046*	
H24C	0.6380	0.5958	−0.2126	0.046*	
H1N	0.7596 (11)	0.414 (2)	0.4823 (17)	0.033 (5)*	

### Atomic displacement parameters (Å^2^)

**Table:**

	*U*^11^	*U*^22^	*U*^33^	*U*^12^	*U*^13^	*U*^23^
O1	0.0361 (5)	0.0295 (6)	0.0207 (5)	0.0038 (4)	0.0064 (4)	−0.0004 (4)
O2	0.0284 (6)	0.0622 (9)	0.0387 (6)	0.0099 (6)	0.0033 (5)	−0.0156 (6)
O3	0.0226 (5)	0.0304 (5)	0.0266 (5)	0.0016 (4)	−0.0001 (4)	−0.0026 (4)
N1	0.0275 (6)	0.0286 (6)	0.0166 (5)	0.0006 (5)	0.0029 (4)	−0.0013 (5)
N2	0.0297 (6)	0.0276 (6)	0.0183 (5)	0.0048 (5)	0.0058 (4)	0.0028 (5)
C1	0.0251 (6)	0.0241 (7)	0.0198 (6)	−0.0004 (5)	0.0040 (5)	0.0022 (5)
C2	0.0279 (7)	0.0352 (8)	0.0196 (6)	0.0020 (6)	0.0007 (5)	0.0001 (6)
C3	0.0255 (7)	0.0353 (8)	0.0253 (7)	0.0036 (6)	0.0007 (6)	0.0006 (6)
C4	0.0301 (7)	0.0331 (8)	0.0273 (7)	0.0089 (6)	0.0034 (6)	0.0000 (6)
C5	0.0277 (7)	0.0246 (7)	0.0193 (6)	0.0008 (5)	0.0060 (5)	0.0028 (5)
C6	0.0231 (6)	0.0229 (6)	0.0187 (6)	0.0008 (5)	0.0033 (5)	0.0023 (5)
C7	0.0238 (6)	0.0218 (6)	0.0174 (6)	−0.0001 (5)	0.0020 (5)	0.0005 (5)
C8	0.0246 (7)	0.0232 (7)	0.0209 (6)	0.0010 (5)	0.0050 (5)	0.0025 (5)
C9	0.0261 (7)	0.0233 (7)	0.0230 (6)	−0.0003 (5)	0.0064 (5)	0.0015 (5)
C10	0.0234 (6)	0.0226 (7)	0.0192 (6)	0.0032 (5)	0.0036 (5)	0.0003 (5)
C11	0.0234 (6)	0.0236 (7)	0.0226 (6)	0.0037 (5)	0.0063 (5)	0.0026 (5)
C12	0.0271 (7)	0.0280 (7)	0.0242 (6)	0.0011 (6)	0.0051 (5)	0.0000 (6)
C13	0.0302 (7)	0.0283 (8)	0.0320 (8)	−0.0017 (6)	0.0062 (6)	−0.0036 (6)
C14	0.0301 (7)	0.0243 (7)	0.0411 (9)	−0.0003 (6)	0.0113 (6)	0.0050 (6)
C15	0.0302 (7)	0.0300 (8)	0.0303 (7)	0.0052 (6)	0.0113 (6)	0.0084 (6)
C16	0.0237 (6)	0.0272 (7)	0.0239 (7)	0.0053 (5)	0.0070 (5)	0.0026 (6)
C17	0.0273 (7)	0.0239 (7)	0.0209 (6)	0.0036 (5)	0.0045 (5)	0.0001 (5)
C18	0.0255 (7)	0.0286 (7)	0.0244 (6)	−0.0002 (6)	0.0052 (5)	0.0013 (6)
C19	0.0220 (7)	0.0335 (8)	0.0334 (8)	0.0026 (6)	−0.0019 (6)	−0.0030 (6)
C20	0.0343 (8)	0.0480 (10)	0.0385 (9)	0.0068 (8)	−0.0056 (7)	−0.0127 (8)
C21	0.0309 (7)	0.0358 (8)	0.0267 (7)	0.0018 (6)	0.0075 (6)	−0.0048 (6)
C22	0.0294 (8)	0.0494 (10)	0.0349 (8)	0.0077 (7)	−0.0025 (6)	0.0011 (8)
C23	0.0295 (7)	0.0459 (10)	0.0399 (9)	−0.0019 (7)	0.0074 (6)	0.0033 (8)
C24	0.0369 (8)	0.0364 (8)	0.0186 (6)	0.0082 (6)	0.0078 (6)	0.0047 (6)

### Geometric parameters (Å, º)

**Table:**

O1—C5	1.2317 (18)	C10—C11	1.441 (2)
O2—C18	1.2115 (19)	C11—C12	1.406 (2)
O3—C18	1.3475 (18)	C11—C16	1.4191 (19)
O3—C19	1.4578 (16)	C12—C13	1.384 (2)
N1—C1	1.3648 (19)	C12—H12A	0.9500
N1—C9	1.3910 (18)	C13—C14	1.405 (2)
N1—H1N	0.86 (2)	C13—H13A	0.9500
N2—C17	1.3716 (19)	C14—C15	1.381 (2)
N2—C16	1.3724 (19)	C14—H14A	0.9500
N2—C24	1.4594 (17)	C15—C16	1.397 (2)
C1—C6	1.3606 (19)	C15—H15A	0.9500
C1—C2	1.5002 (18)	C17—H17A	0.9500
C2—C3	1.533 (2)	C19—C20	1.495 (2)
C2—H2A	0.9900	C19—H19A	0.9900
C2—H2B	0.9900	C19—H19B	0.9900
C3—C4	1.531 (2)	C20—H20A	0.9800
C3—C23	1.531 (2)	C20—H20B	0.9800
C3—C22	1.532 (2)	C20—H20C	0.9800
C4—C5	1.5207 (19)	C21—H21A	0.9800
C4—H4A	0.9900	C21—H21B	0.9800
C4—H4B	0.9900	C21—H21C	0.9800
C5—C6	1.446 (2)	C22—H22A	0.9800
C6—C7	1.5197 (17)	C22—H22B	0.9800
C7—C10	1.5146 (19)	C22—H22C	0.9800
C7—C8	1.5315 (19)	C23—H23A	0.9800
C7—H7A	1.0000	C23—H23B	0.9800
C8—C9	1.355 (2)	C23—H23C	0.9800
C8—C18	1.4781 (19)	C24—H24A	0.9800
C9—C21	1.503 (2)	C24—H24B	0.9800
C10—C17	1.3713 (18)	C24—H24C	0.9800
			
C18—O3—C19	114.36 (11)	C12—C13—C14	121.39 (14)
C1—N1—C9	122.27 (12)	C12—C13—H13A	119.3
C1—N1—H1N	116.4 (13)	C14—C13—H13A	119.3
C9—N1—H1N	115.4 (13)	C15—C14—C13	121.05 (14)
C17—N2—C16	108.41 (12)	C15—C14—H14A	119.5
C17—N2—C24	126.04 (13)	C13—C14—H14A	119.5
C16—N2—C24	125.07 (13)	C14—C15—C16	117.61 (14)
C6—C1—N1	120.71 (12)	C14—C15—H15A	121.2
C6—C1—C2	123.79 (13)	C16—C15—H15A	121.2
N1—C1—C2	115.48 (12)	N2—C16—C15	129.41 (13)
C1—C2—C3	112.70 (12)	N2—C16—C11	108.03 (13)
C1—C2—H2A	109.1	C15—C16—C11	122.51 (14)
C3—C2—H2A	109.1	C10—C17—N2	110.91 (13)
C1—C2—H2B	109.1	C10—C17—H17A	124.5
C3—C2—H2B	109.1	N2—C17—H17A	124.5
H2A—C2—H2B	107.8	O2—C18—O3	121.90 (13)
C4—C3—C23	110.43 (13)	O2—C18—C8	126.06 (14)
C4—C3—C22	110.36 (14)	O3—C18—C8	112.03 (12)
C23—C3—C22	109.28 (14)	O3—C19—C20	108.07 (12)
C4—C3—C2	106.87 (12)	O3—C19—H19A	110.1
C23—C3—C2	111.12 (14)	C20—C19—H19A	110.1
C22—C3—C2	108.75 (13)	O3—C19—H19B	110.1
C5—C4—C3	114.23 (13)	C20—C19—H19B	110.1
C5—C4—H4A	108.7	H19A—C19—H19B	108.4
C3—C4—H4A	108.7	C19—C20—H20A	109.5
C5—C4—H4B	108.7	C19—C20—H20B	109.5
C3—C4—H4B	108.7	H20A—C20—H20B	109.5
H4A—C4—H4B	107.6	C19—C20—H20C	109.5
O1—C5—C6	122.29 (13)	H20A—C20—H20C	109.5
O1—C5—C4	119.05 (13)	H20B—C20—H20C	109.5
C6—C5—C4	118.54 (12)	C9—C21—H21A	109.5
C1—C6—C5	118.99 (12)	C9—C21—H21B	109.5
C1—C6—C7	121.21 (13)	H21A—C21—H21B	109.5
C5—C6—C7	119.73 (12)	C9—C21—H21C	109.5
C10—C7—C6	112.57 (11)	H21A—C21—H21C	109.5
C10—C7—C8	110.43 (11)	H21B—C21—H21C	109.5
C6—C7—C8	109.99 (11)	C3—C22—H22A	109.5
C10—C7—H7A	107.9	C3—C22—H22B	109.5
C6—C7—H7A	107.9	H22A—C22—H22B	109.5
C8—C7—H7A	107.9	C3—C22—H22C	109.5
C9—C8—C18	119.84 (13)	H22A—C22—H22C	109.5
C9—C8—C7	121.71 (12)	H22B—C22—H22C	109.5
C18—C8—C7	118.39 (12)	C3—C23—H23A	109.5
C8—C9—N1	119.58 (13)	C3—C23—H23B	109.5
C8—C9—C21	127.93 (13)	H23A—C23—H23B	109.5
N1—C9—C21	112.48 (12)	C3—C23—H23C	109.5
C17—C10—C11	105.93 (12)	H23A—C23—H23C	109.5
C17—C10—C7	125.43 (13)	H23B—C23—H23C	109.5
C11—C10—C7	128.46 (12)	N2—C24—H24A	109.5
C12—C11—C16	118.31 (13)	N2—C24—H24B	109.5
C12—C11—C10	134.96 (13)	H24A—C24—H24B	109.5
C16—C11—C10	106.70 (12)	N2—C24—H24C	109.5
C13—C12—C11	119.12 (14)	H24A—C24—H24C	109.5
C13—C12—H12A	120.4	H24B—C24—H24C	109.5
C11—C12—H12A	120.4		
			
C9—N1—C1—C6	12.1 (2)	C6—C7—C10—C17	126.79 (14)
C9—N1—C1—C2	−166.63 (13)	C8—C7—C10—C17	−109.86 (15)
C6—C1—C2—C3	23.2 (2)	C6—C7—C10—C11	−58.89 (18)
N1—C1—C2—C3	−158.12 (13)	C8—C7—C10—C11	64.47 (17)
C1—C2—C3—C4	−51.15 (17)	C17—C10—C11—C12	177.98 (16)
C1—C2—C3—C23	69.39 (17)	C7—C10—C11—C12	2.8 (3)
C1—C2—C3—C22	−170.29 (14)	C17—C10—C11—C16	−0.02 (15)
C23—C3—C4—C5	−67.55 (17)	C7—C10—C11—C16	−175.22 (13)
C22—C3—C4—C5	171.52 (13)	C16—C11—C12—C13	1.4 (2)
C2—C3—C4—C5	53.44 (17)	C10—C11—C12—C13	−176.46 (15)
C3—C4—C5—O1	157.14 (14)	C11—C12—C13—C14	−0.3 (2)
C3—C4—C5—C6	−26.6 (2)	C12—C13—C14—C15	−0.4 (2)
N1—C1—C6—C5	−171.47 (13)	C13—C14—C15—C16	0.1 (2)
C2—C1—C6—C5	7.2 (2)	C17—N2—C16—C15	−175.84 (14)
N1—C1—C6—C7	5.7 (2)	C24—N2—C16—C15	−3.4 (2)
C2—C1—C6—C7	−175.67 (13)	C17—N2—C16—C11	1.48 (15)
O1—C5—C6—C1	170.63 (13)	C24—N2—C16—C11	173.94 (13)
C4—C5—C6—C1	−5.5 (2)	C14—C15—C16—N2	178.00 (14)
O1—C5—C6—C7	−6.6 (2)	C14—C15—C16—C11	1.0 (2)
C4—C5—C6—C7	177.32 (13)	C12—C11—C16—N2	−179.29 (12)
C1—C6—C7—C10	103.36 (15)	C10—C11—C16—N2	−0.90 (15)
C5—C6—C7—C10	−79.50 (16)	C12—C11—C16—C15	−1.7 (2)
C1—C6—C7—C8	−20.24 (19)	C10—C11—C16—C15	176.65 (13)
C5—C6—C7—C8	156.90 (12)	C11—C10—C17—N2	0.95 (16)
C10—C7—C8—C9	−104.62 (15)	C7—C10—C17—N2	176.33 (12)
C6—C7—C8—C9	20.22 (19)	C16—N2—C17—C10	−1.55 (16)
C10—C7—C8—C18	72.63 (16)	C24—N2—C17—C10	−173.91 (13)
C6—C7—C8—C18	−162.53 (12)	C19—O3—C18—O2	0.3 (2)
C18—C8—C9—N1	177.22 (13)	C19—O3—C18—C8	−178.99 (12)
C7—C8—C9—N1	−5.6 (2)	C9—C8—C18—O2	11.2 (2)
C18—C8—C9—C21	−1.5 (2)	C7—C8—C18—O2	−166.16 (16)
C7—C8—C9—C21	175.75 (14)	C9—C8—C18—O3	−169.60 (13)
C1—N1—C9—C8	−12.1 (2)	C7—C8—C18—O3	13.10 (18)
C1—N1—C9—C21	166.76 (13)	C18—O3—C19—C20	176.22 (14)

### Hydrogen-bond geometry (Å, º)

**Table:**

*D*—H···*A*	*D*—H	H···*A*	*D*···*A*	*D*—H···*A*
C21—H21*A*···O2	0.98	2.28	2.8073 (19)	113
N1—H1*N*···O1^i^	0.86 (2)	1.98 (2)	2.8161 (15)	163.9 (19)
C24—H24*C*···O2^ii^	0.98	2.60	3.1693 (19)	118

Symmetry codes: (i) *x*, −*y*+1/2, *z*+1/2; (ii) −*x*+1, −*y*+1, −*z*.

## References

[bb1] Agilent (2011). *CrysAlis PRO* and *CrysAlis RED* Agilent Technologies, Yarnton, Oxfordshire, England.

[bb2] Bernstein, J., Davis, R. E., Shimoni, L. & Chang, N.-L. (1995). *Angew. Chem. Int. Ed. Engl.* **34**, 1555–1573.

[bb3] Clark, R. C. & Reid, J. S. (1995). *Acta Cryst.* A**51**, 887–897.

[bb4] Cremer, D. & Pople, J. A. (1975). *J. Am. Chem. Soc.* **97**, 1354–1358.

[bb5] Dolphin, A. C. (2006). *Br. J. Pharmacol.* **147**, 56–62. 10.1038/sj.bjp.0706442 PMC1760727 16402121

[bb6] El-Khouly, A., Öztürk Yildirim, S., Butcher, R. J., Şimsek, R. & Şafak, C. (2012). *Acta Cryst.* E**68**, o3337. 10.1107/S1600536812045886 PMC3588939 23476175

[bb7] Gordeev, M. F., Patel, D. V., England, B. P., Jonnalagadda, S., Combs, J. D. & Gordon, E. M. (1998). *Bioorg. Med. Chem.* **6**, 883–889. 10.1016/s0968-0896(98)00048-0 9730224

[bb8] Gündüz, M. G., Butcher, R. J., Öztürk Yildirim, S., El-Khouly, A., Şafak, C. & Şimşek, R. (2012). *Acta Cryst.* E**68**, o3404–o3405. 10.1107/S1600536812046909 PMC3588994 23476230

[bb9] Öztürk Yildirim, S., Butcher, R. J., El-Khouly, A., Safak, C. & Şimsek, R. (2012). *Acta Cryst.* E**68**, o3365–o3366. 10.1107/S1600536812045722 PMC3588962 23476198

[bb10] Şafak, C. & Şimşek, R. (2006). *Mini Rev. Med. Chem.* **6**, 747–755. 10.2174/138955706777698606 16842124

[bb11] Sheldrick, G. M. (2008). *Acta Cryst.* A**64**, 112–122. 10.1107/S0108767307043930 18156677

[bb12] Triggle, D. J. (2003). *Mini Rev. Med. Chem.* **3**, 215–223. 10.2174/1389557033488141 12570837

[bb13] Triggle, D. J. & Swamy, V. C. (1980). *Chest*, **78**, 174–179. 6249551

[bb14] Vaghy, P. L., Williams, J. S. & Schwartz, A. (1987). *Am. J. Cardiol.* **23**, 9A–17A. 10.1016/0002-9149(87)90170-6 2433927

[bb15] Zamponi, G. W. (1997). *Drug Dev. Res.* **42**, 131–143.

[bb16] Zhou, K., Wang, X.-M., Zhao, Y.-Z., Cao, Y.-X., Fu, Q. & Zhang, S. (2011). *Med. Chem. Res.* **20**, 1325–1330.

